# All Three Endogenous Quinone Species of *Escherichia coli* Are Involved in Controlling the Activity of the Aerobic/Anaerobic Response Regulator ArcA

**DOI:** 10.3389/fmicb.2016.01339

**Published:** 2016-09-07

**Authors:** Johan W. A. van Beilen, Klaas J. Hellingwerf

**Affiliations:** Department of Molecular Microbial Physiology, Swammerdam Institute for Life Sciences, University of Amsterdam Amsterdam, Netherlands

**Keywords:** ubiquinone, menaquinol, naphtoquinones, phos-tag electrophoresis, single-quinone producing mutants, *ubiE*

## Abstract

The enteron *Escherichia coli* is equipped with a branched electron transfer chain that mediates chemiosmotic electron transfer, that drives ATP synthesis. The components of this electron transfer chain couple the oxidation of available electron donors from cellular metabolism (e.g., NADH, succinate, lactate, formate, etc.) to the reduction of electron acceptors like oxygen, nitrate, fumarate, di-methyl-sulfoxide, etc. Three different quinones, i.e., ubiquinone, demethyl-menaquinone and menaquinone, couple the transfer of electrons between the dehydrogenases and reductases/oxidases that constitute this electron transfer chain, whereas, the two-component regulation system ArcB/A regulates gene expression, to allow the organism to adapt itself to the ambient conditions of available electron donors and acceptors. Here, we report that *E. coli* can grow and adjust well to transitions in the availability of oxygen, with any of the three quinones as its single quinone. In all three ‘single-quinone’ *E. coli* strains transitions in the activity of ArcB are observed, as evidenced by changes in the level of phosphorylation of the response regulator ArcA, upon depletion/readmission of oxygen. These results lead us to conclude that all quinol species of *E. coli* can reduce (i.e., activate) the sensor ArcB and all three quinones oxidize (i.e., de-activate) it. These results also confirm our earlier conclusion that demethyl-menaquinone can function in aerobic respiration.

## Introduction

Redox- and phosphoryl-transfer reactions, with coupled chemi-osmotic components, form the core of cellular energetics (Hellingwerf and Konings, 1985). The stepwise release of redox potential in a central electron-transfer system (‘chain’) is fundamental to the vast majority of living cells (Nicholls and Ferguson, 2013). On the other hand (oxygen) radical formation, catalyzed as a side reaction by the same system, is one of the most important causes of damage to the cell (Messner and Imlay, 1999). Therefore, cellular redox reactions need to be tightly regulated. *Escherichia coli* is a facultative (an)aerobic bacterium and it therefore needs to be able to adapt its metabolism, and respiration, to varying oxygen concentrations. Under aerobic conditions, oxygen acts as the preferred terminal electron acceptor (Madigan and Martinko, 2014). When the supply of oxygen becomes limiting, chemically reduced metabolites and electron carriers in their reduced form may accumulate. This ultimately can inhibit growth by oxidative damage (Messner and Imlay, 1999). Under fully anaerobic conditions, alternative terminal electron acceptors, such as nitrate and dimethylsulfoxide (DMSO) may be used to substitute for the role of O_2_ (**Table 1**), and if all of these are absent, the cell may switch to fermentation as a last resort.

**Table 1:** Midpoint potential of various redox couples relevant for this study.

Chemical moiety	Midpoint potential (mV)	Reference
NAD/NADH_2_	-320	Unden and Bongaerts, 1997
MK/MKH_2_	-72	Unden and Bongaerts, 1997
ArcB	-41	Alvarez et al., 2013
Fumarate/succinate	30	Unden and Bongaerts, 1997
DMK/DMKH_2_	36	Unden and Bongaerts, 1997
UQ/UQH_2_	110	Unden and Bongaerts, 1997
Dimethylsulfoxide /DMS^∗^	160	Unden and Bongaerts, 1997
O_2_/H_2_O	820	Unden and Bongaerts, 1997

*^∗^Dimethyl sulfide.*

Accordingly, under both aerobic and anaerobic conditions, *E. coli* manages to proliferate. Anaerobically, a variety of terminal electron acceptors can be used to re-oxidize the NADH pool, generated by the upstream part of central metabolism. The midpoint potential, however, of these alternative electron acceptors is less positive than of the oxygen/water pair (**Table 1**). When requiring alternative electron acceptors, the cell needs to change gears with respect to the respiratory chain and use fewer or smaller steps of redox potential to channel electrons from NADH to the terminal electron acceptor, for the generation of a proton motive force and coupled ATP synthesis, be it that also the magnitude of the proton gradient itself may decrease (Hellingwerf et al., 1981; Tran and Unden, 1998).

The respiratory chain of *E. coli* contains considerable redundancy with respect to the components required for each step of electron transfer from NADH to the terminal electron acceptor (Unden and Bongaerts, 1997). This presumably enables the cell to adjust the metabolic yield of this electron transfer to the needs of the cell, when operating in different metabolic modes. Wild type *E. coli* (including strain MG1655 used in this study) contains several dehydrogenases that oxidize cytoplasmic electron donors, like NADH, and donate electrons to the quinones embedded in the membrane (or quinols when they are present in their reduced form (Sharma et al., 2012). Under aerobic conditions the quinols are then oxidized by any of three terminal oxidases, which transfer electrons to the terminal electron acceptor with slight preference (Sharma et al., 2012). This electron transfer chain has been studied extensively during the past decades, both by others and by ourselves (Van Der Plas et al., 1983; Visser et al., 1984; van Schie et al., 1985; Poole and Cook, 2000; Bekker et al., 2009; Sharma et al., 2012; Steinsiek et al., 2014).

In *E. coli*, there are two primary NADH dehydrogenases (NDH-I and NDH-II) that are part of the standard respiratory chain and that may transfer electrons to any of the three endogenous quinones. In addition, formate dehydrogenase, lactate dehydrogenase, and succinate dehydrogenase may also donate electrons to the quinone pool, thereby oxidizing their respective substrate. The quinone pool of *E. coli* consists of three quinone types, the benzoquinone ubiquinone (UQ) and the naphtoquinones demethylmenaquinone (DMK) and menaquinone (MK); for reviews, see (Unden and Bongaerts, 1997; Sharma et al., 2012), with on average a prenyl side-chain length of 8 (Meganathan, 2001b). Ubiquinone is primarily used during aerobic respiration and nitrite respiration (be it, together with MK; Sharma et al., 2012). Menaquinone is the primary quinone under anaerobic conditions. It is amongst others specifically required for formate dehydrogenase activity and for DMSO (dimethylsulfoxide) reduction (Wissenbach et al., 1990). In agreement with this it has been observed that the total level (i.e., oxidized plus reduced) of the ubiquinones increases, and the total level of the menaquinones decreases, when aerobiosis is gradually increased from 0 to 100% (Bekker et al., 2010). DMK has a rather broad specificity regarding its ability to react with dehydrogenases and reductases/oxidases (Unden and Bongaerts, 1997; Sharma et al., 2012). The three terminal oxidases of *E. coli*, cytochrome *bo* oxidase, cytochrome *bd* I oxidase and cytochrome *bd* II oxidase (Bekker et al., 2009), are somewhat specific in terms of the quinol electron donor that they prefer, one of the determining factors being their mutual redox midpoint potential (Unden and Bongaerts, 1997), but all three can oxidize both ubiquinol and demethylmenaquinol (Sharma et al., 2012). Other terminal electron acceptors use dedicated enzymes such as nitrate (NarGHI), fumarate (FrdABCD), DMSO (DmsABC), TMAO (trimethylamine *N*-oxide; TorCDA), and nitrite (NrfABCD), each with their own quinone specificity (Unden and Bongaerts, 1997). A final complicating factor in understanding specificity in electron transfer reactions in the electron transfer chain of *E. coli* are the reactions between reduced and oxidized species of the different quinones. Although very little solid data on this aspect is available, our previous studies indicated that this aspect cannot be ignored (Bekker et al., 2010).

For any *E. coli* cell living in the ambient environment, the transition from anaerobic to aerobic conditions and vice versa is one of the most thoroughgoing transitions. Going through this transition requires alteration of the functional activity of a large number of metabolic- and respiratory pathways, to the extent that the transcription of some 200 genes has to be adjusted in this transition (Salmon et al., 2005). A large part of this transcriptional regulation is carried out by the Anoxic Redox Control (Arc) two-component system, that functions complementary to three additional systems: FNR, SoxRS, and OxyR (for a review see Iuchi and Weiner, 1996). The Arc two-component system consists of the classical response regulator ArcA, and the redox-sensitive, membrane-embedded kinase, ArcB. ArcA can both be phosphorylated (leading to formation of the transcriptionally active state) and dephosphorylated (its inactive state) by ArcB.

The signal-induced switching of the activity of ArcB, between its kinase- and phosphatase function, is the subject of this study. After an early consensus that it could not be molecular oxygen that is the signal for ArcB, Georgellis et al. (2001) convincingly showed that *in vitro*, UQ_0_ inhibits the autophosphorylation of ArcB – and by inference its kinase activity – and UQ_0_H_2_ actives it. With Alexeeva et al. (2002) we introduced a system that allows titration of the rate of electron transfer to oxygen in chemostat-grown cells of *E. coli* – the aerobiosis system. If it would be only the redox state of the ubiquinone pool that would regulate the activity of the ArcB sensor one would predict that in this aerobiosis system a sigmoidal relation would be obtained between ArcB activity and the degree of aerobiosis (reflecting the gradual transition of predominance of ubiquinol/ubiquinone). We did observe, however, a relation that was more complex (Alexeeva et al., 2002; Bekker et al., 2010), and interpreted this result as a consequence of the fact that not only ubiquinol but also (dimethyl)menaquinol would be able to reduce, and hence activate, ArcB. Conversely, other authors have found different relations between aerobiosis and ArcB activity (e.g., Rolfe et al., 2011; Alvarez et al., 2013; Steinsiek et al., 2014)

Although, meanwhile we have provided more evidence supporting our interpretation (Sharma et al., 2013), Alvarez et al. (2013) have published an updated model for the regulation of ArcB activity by the quinone pool of *E. coli*, based on an *in vivo* determination of the redox midpoint potential of the redox-sensitive active site cysteine(s) residue of ArcB. Using the system of cysteine/cystine redox buffers a value of -41 mV was obtained for one (or both) of the critical cysteines that have been assigned to this role. They went then on to argue that it would be logical to assume that ubiquinone (with a midpoint potential of +100 mV) can only inactivate ArcB (and by inference: ubiquinol cannot activate it), although we have clearly demonstrated (amongst others through the use of so-called UQ-only mutants) that both ubiquinol and menaquinol can both activate ArcB in *E. coli* (Bekker et al., 2010; Sharma et al., 2012). Nevertheless, Alvarez et al. (2013) postulate their ‘yin and yang’ model for ArcB regulation: exclusive activation of ArcB by menaquinol (midpoint potential: -74 mV) and exclusive inactivation by ubiquinone.

The laws of thermodynamics, however, hold that whether or not two redox active molecules will react – besides chemical specificity – is not so much dictated by their redox midpoint potential, but rather by the actual redox potential of the two couples involved (Berg et al., 2002). Therefore, there is no compelling *a priori* reason to assume that high concentrations of ubiquinol would not be able to activate ArcB, nor high concentrations of menaquinone to de-activate it, particularly if activation of a subset of ArcB molecules would suffice to increase the net phosphorylation level of ArcA. Therefore, we have constructed three mutants of *E. coli* that contain a single quinone type only, and studied ArcB regulation in these strains. Consistent with our previous observations, here we report that all three ‘single-quinone’ mutants display the ArcB activation/deactivation cycle.

For the construction of the menaquinone-only mutant we cloned the *ubiE* homolog *menH* from *Bacillus subtilis* in *E. coli*. The resulting mutant strain turned out to convert essentially all its demethylmenaquinone/ol into menaquinone/ol.

## Materials and Methods

### Strains Used in this Investigation

All strains used in this study are derived from the *E. coli* K12 wild type strain MG1655 (**Table 2**). This strain contains all three biologically active quinones that are known to be present in *E. coli*. The deletion mutants AV34 and AV36, containing only UQ and only DMK, respectively, were obtained from our own strain collection (Sharma et al., 2013). The DMK-only and MK-only strains were constructed using the Gene Doctoring system (Lee et al., 2009). Plasmids were designed and constructed based on pDOC-K, to delete *ubiCA* or introduce *ubiE* or *menH_Bsu_* and combinations of these, using a spectinomycin resistance cassette (-S) from pDG1661 (Guérout-Fleury et al., 1996) instead of the kanamycin resistance cassette (-K). The *ubiE* gene or its homolog *menH* from *B. subtilis* were genomically integrated in the *yoeG* locus, which we assumed to be a neutral site, by using pDOC-K-yoeG-ParaMenH [note that *yoeG* is annotated as the defective integrase of the CP4-44 prophage (Wang et al., 2010)]. For all plasmids, 250 bp of the 3′ and 5′ ends of the target locus were cloned into the pDOC vector, to allow homologous replacement with the resistance cassette (and additional genes when present). The arabinose inducible promotor used (P_ara_) was PCR amplified from pACBSCE and fused to the downstream gene with an overlap-extension PCR. For overexpression of *menH*_Bsu_, 0.1% arabinose (w/v) was used.

**Table 2:** Strain and plasmids used in this study.

Name	Genotype	Reference
*E. coli* MG1655	K-12 wild type	Lab stock
*E. coli* AV34	*ΔmenA::kan*	Sharma et al., 2013
*E. coli* AV36	*ΔubiE::kan*	Sharma et al., 2013
*E. coli* DMK-only	*ΔubiE::kan ΔubiCA::Spc*	This study
*E. coli* MK-only	*ΔubiCA::Spc ΔyoeG:: Kan P_ara_-menH_Bsu_*	This study
pACBSCE	[see reference]	Lee et al., 2009
pDOC-K	[see reference]	Lee et al., 2009
pDOC-K-yoeG-ParaMenH	See text, Supplementary Figure S1A	This study
pDOC-S-ubiCA	See text, Supplementary Figure S1B	This study

It is important to note that selection of MK-only *E. coli* mutants was successful only (in our hands) when LB-agar plates, containing 20 mM glucose and 0.2 mM UQ_0,_ were used under strict anaerobic conditions, in an anaerobic jar flushed with N_2_ and containing an AnaeroGen pack (Oxoid, Thermo Scientific) to eliminate remaining traces of O_2_. When cultured under (micro)aerobic conditions for longer periods of time, multiple revertant mutants could be picked up, containing DMK only or DMK + MK, which in liquid cultures outcompete the MK-only strain.

### Growth of the *E. coli* Strains in Batch Culture

All strains were inoculated from single colonies and pre-cultured in LB medium containing 20 mM glucose and 50 mM DMSO (dimethylsulfoxide) at 37°C under continuous agitation with anaerobic conditions in a jar flushed with nitrogen gas. This culture was used as a 0.1% inoculum for 25 ml cell cultures which were grown as anaerobic batch culture at 37°C using Evans’ salt medium with nitrilo-acetic acid (2 mM) and sodium phosphate buffer (100 mM, pH 7) to increase buffering capacity (Evans et al., 1970; Sharma et al., 2013). Glucose (20 mM) was used as carbon source and 50 mM DMSO was used as terminal electron acceptor. For anaerobic transition experiments, the contents of these were then transferred to a batch fermenter containing 500 ml of Evans’ medium containing 20 mM glucose, no extra DMSO and continuous nitrogen gas sparging at a flow rate of 50–80 ml/min to maintain anaerobic conditions. These anaerobic cultures, were used for the anaerobic/aerobic transition experiments, keeping all the other conditions the same. All strains were assessed in at least three biologically independent replicates.

### Quinone Extraction and Analysis

The extraction and analysis of quinones was carried out essentially as described by Bekker et al. (2007). Briefly, at each time point a 2 ml sample was taken in 6 ml of a 1:1 (v/v) mixture of ice-cold methanol and petroleum ether (evaporation temperature 40–60°C). The mixture then was vortexed for 1 min and centrifuged at 3,000 rpm for 1 min. Then the upper petroleum ether phase was transferred to a glass tube under a nitrogen atmosphere and containing 80 μl 1-hexanol. After evaporation of the petroleum ether (20–30 min), the 1-hexanol was transferred to a glass high-performance liquid chromatography (HPLC) vial and stored at -20°C until analysis within 48 h.

The samples were fractionated with HPLC using a reversed-phase Lichrosorb (Chrompack, Bergen op Zoom, The Netherlands) RP10 C18 column (size, 4.6 mm; internal diameter, 250 mm). The column was equilibrated with pure methanol as the mobile phase at the flow rate of 2 ml/min. Detection of the quinones was performed using a UV/Vis absorption detector at 290 nm for ubiquinone (UQ) and at 248 nm for naphtoquinones (DMK and MK). All reduced quinone species were also detected with an Agilent 1200 series fluorescence detector, coupled in series with a UV/Vis detector, using 238 and 375 nm as the excitation- and emission wavelength, respectively, with the photomultiplier gain set to 12. The amount of each quinone species was calculated from the relevant area under the peak.

Standards of all (oxidized) quinone species were prepared by isolating relevant fractions from the HPLC and re-extracting these as described above. Concentrated and purified quinones were analyzed on a UV/Vis spectrophotometer and based on their respective extinction coefficient (White, 1965; Holländer, 1976), their concentrations were determined. The UQ_8_ used in some experiments to assist the respiratory chain of the MK only strain were obtained as described above, from cultures of *E. coli* AV34.

### Measurement of ArcA Phosphorylation with Phos-tag Electrophoresis

Relative ArcA phosphorylation levels were measured with Phos-tag^TM^-acrylamide gel electrophoresis and Western immunoblotting as described by Rolfe et al. (2011). The purified ArcA and antibodies were obtained as described (Bekker et al., 2010). *In vitro* phosphorylation was achieved by incubation of ArcA in 30 mM HEPES pH 7.5, 10 mM MgCl_2_, 25 mM acetyl phosphate and 10% glycerol. All samples were taken from at least three biologically independent replicates, sampled in technical duplicates.

### Metabolite Analysis

Samples from each time point were processed for HPLC analysis essentially as described before (Sharma et al., 2012); a 1 ml sample was mixed with 100 μl 35% perchloric acid and subsequently 55 μl 7 M KOH was added. Filtered supernatants were analyzed for glucose consumption and fermentation products. Glucose, pyruvate, lactate, formate, acetate, succinate, and ethanol contents were determined by HPLC (LKB and Pharmacia, Oregon City, OR, USA) using a REZEX organic acid analysis column (Phenomenex, Torrance, CA, USA) at 45°C, with 7.2 mm H_2_SO_4_ as the eluent, using an RI 1530 refractive index detector (Jasco, Easton, MD, USA) and AZUR chromatography software (St. Martin D’Heres, France) for data analysis.

## Results

### Construction of Three ‘Single Quinone’ *E. coli* Strains

**FIGURE 1:**
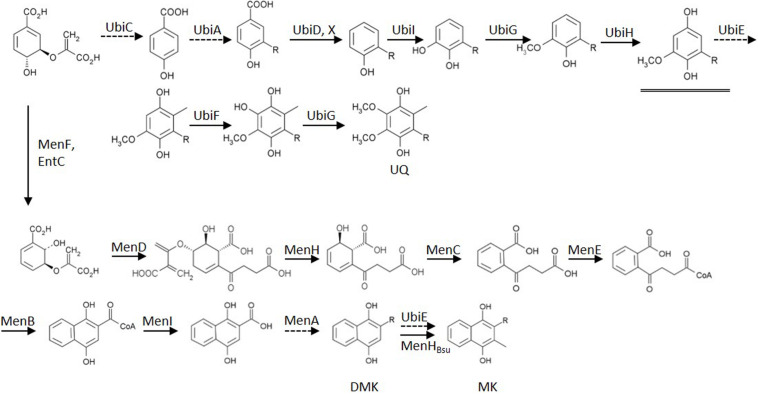
**Biosynthesis routes of the quinones of *Escherichia coli*, starting from chorismate**. Dashed arrows indicate enzymes deleted in mutants used in this study. The double arrow symbolizes the action of the introduced heterologous MenHBsu. The underlined intermediate C1-demethyl-C6-demethoxy-Q8 (DDMQ8) may accumulate in a ubiE mutant strain (for references: see text). Established bio-active quinones are indicated via their abbreviation. UQ, ubiquinone; DMK, demethylmenaquinone; MK, menaquinone; R, isoprenoid sidechain.

**FIGURE 2:**
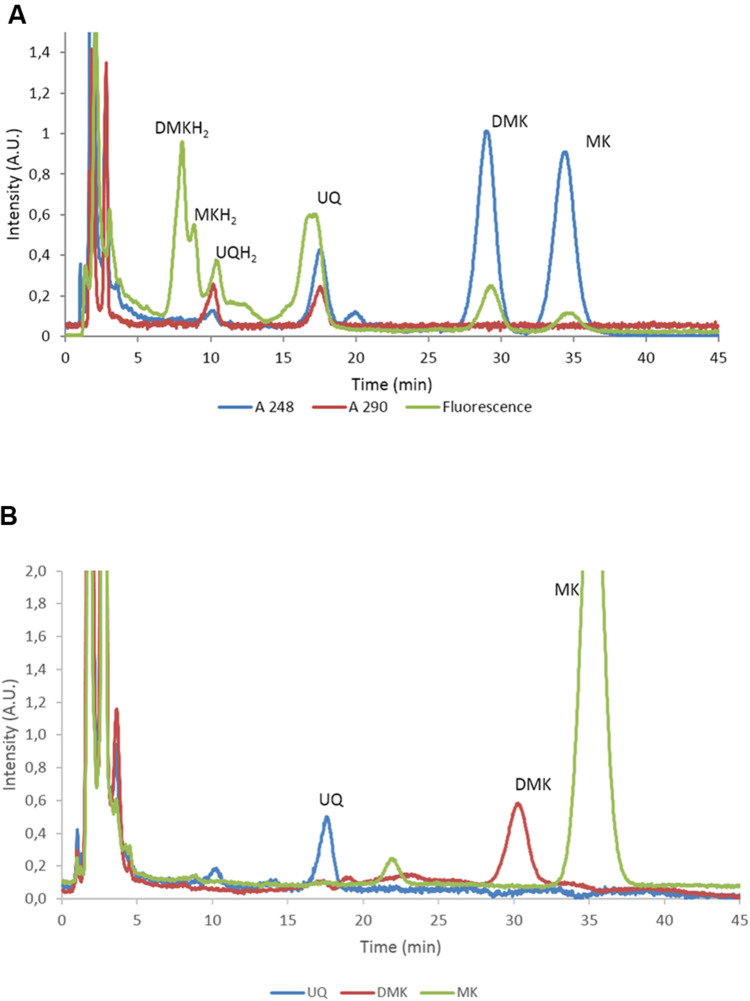
**Separation and quantification of the main quinone types of *E. coli*. (A)** High-performance liquid chromatography (HPLC) trace of *E. coli* MG1655 with the eluate analyzed with A_248_, A_290_ and fluorescence. The identified quinones are indicated. UQ, ubiquinone; DMK, demethylmenaquinone; MK, menaquinone. Note that UQ is not fluorescent at these wavelengths and that the peak in the fluorescence channel at approximately 17 min. does not correspond to UQ. **(B)** HPLC traces recorded at 248 nm of *E. coli* strains AV34, DMK-only (AV36), MK-only (i.e., *E. coli ΔubiCA* complemented with *menH* from *Bacillus subtilis*. UQ, ubiquinone; DMK, demethylmenaquinone; MK, menaquinone.

To characterize the role of the various quinones of *E. coli* in the regulation of the activity of two-component kinase/phosphatase ArcB, we set out to construct a set of *E. coli* strains that have only one single quinone species. In *E. coli*, three dominant quinone types are found that are active in facilitating electron transfer in the respiratory chain (Sharma et al., 2012). These are the benzoquinone ubiquinone (UQ) and two naphtoquinones, demethylmenaquinone (DMK) and menaquinone (MK; see also **Figure 1**). From a batch culture of wild type *E. coli* MG1655, both the oxidized and reduced form of all three types of quinone can be identified, as is shown in **Figure 2A**. We note, however, that without specific precautions the oxidized/reduced (ox/red) ratio observed in such samples for the menaquinones does not accurately reflect their *in vivo* ox/red ratio because of the rapid spontaneous auto-oxidation, in contrast to the observations made with ubiquinone (Bekker et al., 2010; Sharma et al., 2013). Furthermore, for optimal quantification of these six species three different detector settings are required, for absorption at 248 and at 290 nm and for fluorescence emission, respectively.

Strains were available from previous studies that contain as their sole quinone either ubiquinone (i.e., AV34) or demethyl-menaquinone (AV36; Sharma et al., 2013). DMK is the direct precursor of MK and its methylation is catalyzed by UbiE (Lee et al., 1997). A knock-out of only *ubiE*, however, would lead to accumulation of C1-demethyl-C6-demethoxy-Q_8_ (DDMQ; see **Figure 1**). DDMQ most probably is redox-active (our unpublished results and Aussel et al., 2014) and might substitute for UQ, together with DMK in electron transfer reactions in the electron transfer chain. Therefore a *ΔubiE* strain would not be suitable for proper analysis of the role of DMK. Instead, it is important to use a *ΔubiE ΔubiCA* double mutant for such experiments. Such a mutant strain was constructed by knocking out *ubiCA* in AV36 (*ΔubiE*). This was achieved via the use of plasmid pDOC-S-ubiCA and the resulting strain is referred to as the ‘DMK only’ strain in the remainder of this communication.

An *E. coli* strain with MK as its sole quinone cannot be created by a gene knock-out only. The alternative approach that we initially selected, i.e., overexpression of *ubiE* in a Δ*ubiCA* mutant strain (i.e., a strain that does not contain ubiquinone) did not result in complete conversion of DMK to MK (our unpublished results). The Gram-positive soil-dwelling bacterium *B. subtilis* uses MK as the sole quinone in its respiratory chain. Because the biosynthesis pathway of MK in *B. subtilis* is very similar to that of *E. coli*, we decided to overexpress the UbiE homolog MenH from *B. subtilis* in *E. coli* MG1655 and in its Δ*ubiCA* derivative strain.

As can be seen in **Figure 2B**, induced expression of a genome-integrated copy of *menH* completely eliminated the presence of DMK in the mutant strain. Subsequent long term cultivation of this MK-only strain under aerobic conditions resulted in the emergence of revertant strains that readily out-competed the original mutants via a mutation that altered expression of one or both of the methyltransferases (i.e., giving rise to a DMK plus MK- or even DMK-only phenotype; our unpublished results). However, anaerobic cultivation with DMSO as the terminal electron acceptor resulted in dense cultures within 48 h with a stable, MK-only phenotype (**Figure 2B**). **Figure 2B** also confirms the phenotype of the other two single quinone strains. The components visible in the MK only strain eluting at 22 min, and the fluorescent component in MG1655 eluting at 17 min. have not been further identified.

### The Phosphorylation State of ArcA

**FIGURE 3:**
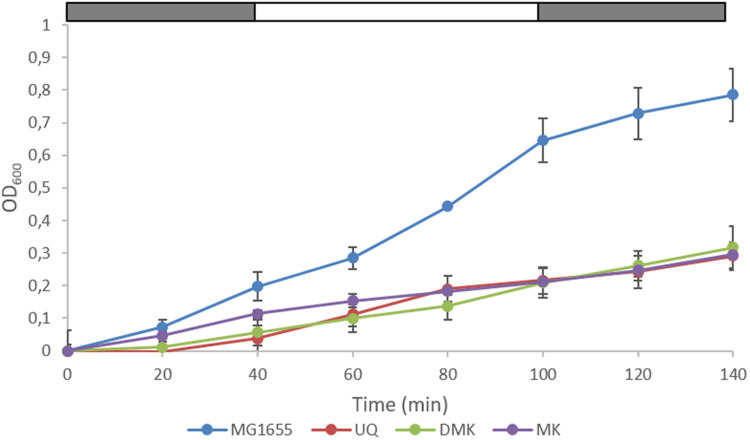
**Growth of wild type *E. coli* and the three single quinone strains in (an)aerobic transition experiments.** The last three data points correspond to anaerobic growth, except the MK-only strain for which the last two points are anaerobic. The bar above each panel indicates sparging with N_2_ (gray) or air (white). Data represent the average of three biologically independent replicates, error bars indicate the standard deviation.

**FIGURE 4:**
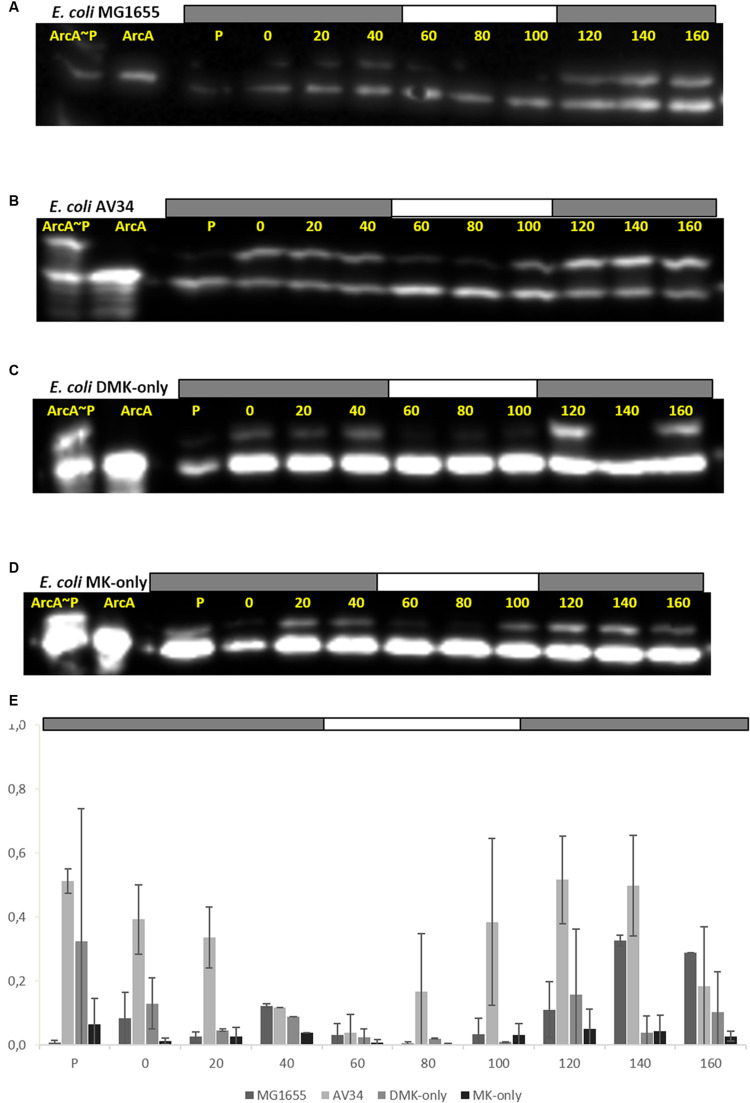
**All quinones of *E. coli* can both reduce (activate) and oxidize (inactivate) ArcAB/A.** ArcA∼P Phos-Tag Western Blot of *E. coli* strain used in this study. The lower band represents ArcA while the upper band represents ArcA∼P, which is slowed down by the Phos-Tag gel during electorphoresis. The first two lanes are reserved for the ArcA standard, and *in-vitro* phosphorylated ArcA, respectively. P indicates the pre-culture and the other lanes the sampling time (min). The bar on top of **(A–D)** indicates N_2_ (gray) or air (white) sparging. **(A)** *E. coli* MG1655, **(B)** *E. coli* AV34 (UQ-only), **(C)** *E. coli* DMK-only, **(D)** *E. coli* MK-only, **(E)** Graph of the phosphorylation level of ArcA in the different strains used in this study. The bar graphs shows the results of densitometric quantitation of the ArcA∼P/ArcA ratio. Data represent the average of three biologically independent replicates, error bars indicate the standard deviation.

We next carried out a series of (an)aerobic transition experiments with the wild type strain and the three single quinone strains (**Figure 3**). The strains were pre-cultured under anaerobic conditions in Evans’ medium, with glucose as the carbon and energy source, and subsequently transferred to shake-flasks and flushed with N_2_. After a few hours of growth, the cultures were switched from sparging with N_2_ to sparging with compressed air (60 min), and then back to N_2_ (60 min), during which samples were taken. It can be seen that the growth rate of all three single quinone strains is reduced compared to that of the wild type strain. Cells sampled from these cultures were then assessed for the phosphorylation state of ArcA (**Figure 4**) and the redox state of their (ubi)quinone pool (Supplementary Figure S2).

The anaerobic- to aerobic shift assay with MG1655 shows that both kinase as well as phosphatase activity of ArcB can be observed (**Figure 4A**). However, during the first 40 min of the experiment generally a lower level of ArcA∼P was observed than the level shortly after switching back to anoxic conditions. This is consistent with earlier experiments which showed highest UQH_2_/UQ ratios immediately upon anaerobiosis (Bekker et al., 2007). The UQ-only strain AV34 had a far stronger ArcA∼P signal under anoxic conditions and this signal quickly disappeared when the cells were exposed to oxygen (**Figure 4B**). This shows that ubiquinone is able to facilitate the transition of ArcB’s activity in both directions, i.e., UQH_2_ can activate and UQ can inactivate ArcB, contrasting the claim that UQH_2_ cannot activate this kinase because of presumed incompatibility of the respective midpoint potential values.

In the DMK-only strain, ArcA∼P could also be detected. However, the level of phosphorylated ArcA in this strain is lower than in the wild type and in AV34 (the UQ-only strain), but here too, a clear trend during the anarobiosis/aerobiosis transition could be observed (**Figure 4C**). This is possibly due to ArcB’s preference for ubi- rather than menaquinols, and the lower midpoint potential of DMK compared to UQ, which may decrease the rate of electron transfer from DMKH_2_ to ArcB. A similar observation was made in the MK-only strain, where very little ArcA∼P was observed in our anaerobic/aerobic shift assay (**Figure 4D**).

The MK-only strain showed the lowest level of ArcA∼P, but the anaerobic/aerobic/anaerobic trend is clearly visible. These results demonstrate that all three quinone species are able to control the activity or ArcB.

### The Redox State/Potential of the (Ubi)quinone Pool

The quinone pool redox state is considered to be the main regulatory input signal for ArcB-kinase activity, by regulating its switching between kinase and phosphatase activity. In order to link the Q-pool redox state with the ArcA phosphorylation state directly, the Q-pool redox state was measured using an HPLC setup as described before (Bekker et al., 2007; Sharma et al., 2013). Reduced UQ and DMK appeared to be quite stable in hexanol at room temperature for the time period necessary to extract and isolate these quinones. However, MKH_2_ (auto)oxidized very rapidly. The data presented here on the MK redox state (Supplementary Figure S2) can therefore not be considered to represent the *in vivo* state.

In the wild type organism, the UQ/UQH_2_ ratio seems to follow the anaerobic/aerobic shift reasonably well, being more reduced under anaerobic conditions and becoming oxidized when oxygen is present (Supplementary Figure S2A). Both DMK and MK are more reduced under initial anaerobic conditions, but as these pools are more rapidly oxidized and less rapidly reduced, no clear peaks are seen with the later samples (e.g., Supplementary Figure S2B). Strikingly, under aerobic conditions, the level of MK dropped below the detection limit in some samples. As soon as anaerobic conditions returned, MK levels rose again.

### Effect of the Quinone Composition on the Pattern of Fermentation Products Formed in the Three Single- Quinone Mutants during (An)aerobic Transitions

Because metabolic end-products reflect the cell’s physiology, the remaining extracellular glucose and several additional exo-metabolites were analyzed quantitatively with HPLC. As the phosphorylation state of ArcA modulates the expression of several of the respiratory chain components and TCA-cycle enzymes, the degree of activation (i.e., phosphorylation state) of this response regulator might also be inferred from the composition of these metabolic end products.

During anaerobic growth (and also in the pre-culture; data not shown), considerably more formic acid is formed by all three single quinone strains, as compared to the wild type organism (Supplementary Figure S3). This formic acid is rapidly consumed in both MG1655 and AV34 (the UQ only strain) under aerobic conditions. When these strains are switched back to anaerobiosis, formic acid production only slowly resumes. The two menaquinone containing strains, under aerobic conditions, do not appear to catabolize the formic acid produced.

Besides this, MG1655 and AV34 mainly secrete acetate as a result of overflow metabolism (Supplementary Figures S3A,B). Strain AV34 (UQ-only) secretes predominantly acetate under glucose-excess conditions. Strikingly in the two naphtoquinone-only producing strains, i.e., DMK-only, but especially the MK-only strain, fermentation is mostly directed toward lactate production, thus preventing NAD^+^ depletion.

## Discussion

### Construction of the Single-Naphtoquinone Containing Strains of *E. coli*

We set out to investigate the signals inducing transitions of the activity of ArcB, between its kinase- and phosphatase function. Rather than modulating the concentration of all six quinone species in wild type strains (see **Figure 2A**) we choose to construct mutants that contain the oxidized and reduced form of a single quinone only. From previous work both a UQ only (AV34) and a ‘DMK only’ strain (AV36) were available (Sharma et al., 2013). The latter, however, was constructed in such a way that there was considerable risk that a redox-active intermediate, C1-demethyl-C6-demethoxy-Q_8_ (DDMQ), with a midpoint potential likely more positive than UQ) would accumulate, which then also might become involved in the (de)activation of ArcB. We therefore constructed an improved version of the DMK only strain and a strain containing exclusively MK as its quinone.

For construction of this latter strain it is relevant to know that, in contrast to *E. coli*, *B. subtilis* uses only MK in its respiratory chain, and that this organism also possesses a cytochrome *c* oxidase branch in addition to its quinone oxidase branch (Poole and Cook, 2000; Winstedt and von Wachenfeldt, 2000) in its electron transfer chain. Intriguingly, *B. subtilis* has a stronger preference for aerobic conditions than *E. coli* does (Nakano and Zuber, 1998). Despite the fact that the route of synthesis of MK is similar in both organisms, no DMK has been observed to be present in *B. subtilis*, nor is this naphtoquinone assumed to have a biological function in this Gram-positive organism (Kröger and Dadák, 1969; Taber et al., 1981). This prompted us to try the *B. subtilis* homolog of UbiE, MenH_Bsu_, to convert DMK to MK in *E. coli* (Lee et al., 1997). This resulted indeed in very efficient conversion of DMK, which fully depleted the DMK-pool of *E. coli* and allowed the construction of an MK-only *E. coli* strain, as was shown by analysis of its quinone complement (**Figure 2B**). The latter figure also confirms the phenotype of the DMK only strain.

The observation that this improved DMK only strain (i.e., a strain in which DDMQ no longer can accumulate), grows reasonably well also aerobically (see **Figure 3** and JWAvB and KJH, unpublished observations) is also relevant because of “*the classic view associating ubiquinone to aerobic growth and menaquinone to anaerobic growth*” (Aussel et al., 2014): Presumably at least one of the three terminal oxidases of *E. coli* has significant affinity for demethyl-menaquinol. Whether or not the same holds for menaquinol cannot be concluded because of the high frequency with which suppressor mutants appear (see Construction of Three ‘Single Quinone’ *E. coli* Strains) under the conditions tested. This mutation frequency might decrease micro-aerobically, as it is possible that reactive oxygen species (ROS) are formed under aerobic conditions that can cause DNA damage (Iuchi and Weiner, 1996).

### Quinone Specificity of Signal Input into the Two-Component Sensor ArcB

It is now generally accepted that ubiquinone inhibits the kinase activity of ArcB and therefore activates its phosphatase function (10). However, whether the other quinones of *E. coli* are capable of doing so has been debated. Based on the relation between the rate of oxygen consumption and ArcB/A activity we proposed in 2010 that besides ubiquinone, also a naphtoquinone must be able to switch ArcB into its phosphatase function (Sharma et al., 2012).

Although, meanwhile we have provided more evidence supporting our interpretation (Sharma et al., 2013), Georgellis et al. have published an updated model for the regulation of ArcB activity by the quinone pools of *E. coli*, based on an *in vivo* determination of the redox midpoint potential of the redox-sensitive active site cysteine(s) residue of ArcB (Alvarez et al., 2013). They propose that ubiquinone (with a midpoint potential of +100 mV) can only inactivate ArcB, because ubiquinol would not be able to transfer electrons to the cysteines of ArcB. This latter conclusion was presumably also proposed based on earlier work in which it was shown that UQ_0_H_2_ and menadione *in vitro* cannot activate ArcB (for review see Malpica et al., 2006). According to their interpretation only the two naphtoquinols can activate ArcB (13). However, through the use of a so-called single quinone mutants we had shown that both ubiquinol and presumably also menaquinol can activate ArcB in *E. coli* (Bekker et al., 2010; Sharma et al., 2012).

We here show that all three types of *E. coli* cells with a linear respiratory chain at the quinone level are capable of regulating (i.e., activating and deactivating) the activity ArcB, that is, in all three single-quinone strains, the ArcA/ArcA∼P ratio responds to modulation of the redox state of the respective quinone pool through variation of oxygen availability. For both naphtoquinone-containing strains this is new evidence; for the DMK only strain because we can now exclude that the biosynthetic intermediate C1-demethyl-C6-demethoxy-Q_8_ might have been responsible for modulation of ArcB activity in this strain instead (see also above).

In our anaerobic/aerobic transition experiments, low levels of ArcA∼P were found in the (D)MK-only strains. Presumably, residual amounts of oxygen and/or DMSO are still being reduced, which helps to keep the respective quinone pool in a partially oxidized state. Here, it is also relevant to note that SixA may inhibit the kinase activity of ArcB when alternative electron acceptors are available (Matsubara and Mizuno, 2000). Nevertheless, also long-term anaerobic incubation showed clearly that the ArcB/A system is functional and ArcA can be phosphorylated *in vivo* in these strains (our unpublished results). These results re-enforce the interpretations made in our earlier studies (6, 8). In these interpretations the first-order assumption would be that all three quinols activate ArcB with the same rate. At this stage it seems that this assumption does not fully hold. But then again of course rates of ArcB activation are not only determined by quinol specificity, but also by their concentration which are partly determined via the redox potential of the respective pools.

### Effect of Single Quinone Mutations on the Fermentation Products Produced during (An)aerobic Transitions in Batch Cultures of *E. coli*

Several notable effects can be observed in the pattern of formation of fermentation products in the wild type and in the three single quinone mutants in batch cultures undergoing (an)aerobiosis transitions (Supplementary Figure S3). Glucose is indeed much faster consumed in the wild type than in the three mutants, consistent with the growth rate of the four strains (**Figure 3**), be it that a significant part of it is converted into acetate and ethanol via overflow metabolism (Liao and Farmer, 1997; Picon et al., 2005). Succinate production is considerably lowered in the two naphtoquinone-only mutants, as compared to the wild type strain, consistent with the fact that the fumarate reductase enzyme is specific for menaquinone (Wallace and Young, 1977). However, this lowered succinate production is also observed in the UQ only mutant (Supplementary Figure S3B). Formate does not appear to be degraded by the DMK and MK–only strains, in agreement with earlier reports (Wallace and Young, 1977). The most striking difference between the four strains is the high rate of lactate formation, which goes at the cost of formation of the other fermentation products, in the two mutants that cannot make ubiquinone. This might confirm that the respiratory lactate dehydrogenase (LdhA) is highly specific for ubiquinone as the electron acceptor, as it would produce D-lactate (Futai, 1973; Wallace and Young, 1977; Matsushita and Kaback, 1986). However, the methylglyoxal pathway could produce significant amounts of lactate (L+D), which would bypass central metabolism completely. To what extent this shunt is used is beyond the scope of this investigation. There has been speculation in the literature that specific fermentation products, e.g., D-lactate, might also modulate the activity of ArcB (Georgellis et al., 1999; Rodriguez et al., 2004), without, however, proposing a mechanism for this. We think that the current results do not provide incentives to try and propose such a mechanism (Malpica et al., 2006).

### Measurement of the Redox State of the Pools of the Naphtoquinones

In experiments with *Bacillus megaterium*, the menaquinone pool has been observed to be in a highly reduced state (up to 85% reduced) when cells were grown anaerobically, while in the same cells under aerobic conditions this pool was found to be only 10% reduced (Kröger et al., 1971). However, to obtain these results an approach is required that uses specialized equipment; and this approach is only suitable for species with a single type of quinone. DMK too has been assessed in a similar fashion, in *Hemophilus parainfluenzae* (White, 1965). These levels for MK and DMK are not very different from what has been observed in *E. coli* for UQ (Bekker et al., 2007). It would be very interesting to find out whether DMK and MK can be reduced to a similarly high level in *E. coli*, but such experiments are technically very challenging. It is relevant to note that with a similar procedure as the one used here, plus extra precautions for very rapid analysis, it was possible to analyze the *in vivo* redox state of plastoquinone in *Synechocystis* sp. PCC6803 (Schuurmans et al., 2014). We do anticipate, however, that the data on the *in vivo* amount of reduced naphtoquinones reported in this contribution represent a gross underestimate due to the rapid autooxidation of these quinones.

### Outlook

To understand the modulation of the activity of ArcB in even more detail multi-omics analyses will be necessary, similar to the ones recently published for glucose repression (Borirak et al., 2015). That approach provides a more detailed picture of the metabolic consequences of ArcB (de)activation, and also an independent and complementary way to assay this via transcript profiling (e.g., Wareham et al., 2016). In such future experiments it will be an asset to be able to modulate the size of the proton motive force independently and orthogonally, e.g., with a light-dependent proton pump like proteorhodopsin, because of the recent report that that will allow an independent modulation of the rate of formation of ROS (see Na et al., 2015). Such an approach would then also allow for an independent test whether the size of the proton motive force and ROS formation can or cannot directly modulate the activity of ArcB (Bogachev et al., 1995; Iuchi and Weiner, 1996; Malpica et al., 2004).

## Author Contributions

JvB and KH jointly designed the study, JvB carried out the experiments, and KH and JvB jointly interpreted the results and wrote the paper.

## Funding

This work was supported by the SysMo-SUMO2 project, a European Transnational Funding and Research Initiative.

## Conflict of Interest Statement

The authors declare that the research was conducted in the absence of any commercial or financial relationships that could be construed as a potential conflict of interest.
