# Corrigendum: All Three Endogenous Quinone Species of *Escherichia coli* Are Involved in Controlling the Activity of the Aerobic/Anaerobic Response Regulator ArcA

**DOI:** 10.3389/fmicb.2020.01503

**Published:** 2020-07-15

**Authors:** Johan W. A. van Beilen, Klaas J. Hellingwerf

**Affiliations:** Department of Molecular Microbial Physiology, Swammerdam Institute for Life Sciences, University of Amsterdam, Amsterdam, Netherlands

**Keywords:** ubiquinone, menaquinol, naphtoquinones, phos-tag electrophoresis, single-quinone producing mutants, *ubiE*

In the original article, there was a mistake in the legend for Figure 1 as published. There were small discrepancies between the text of this legend and the drawing of the figure. The correct legend appears below.

In the original article, there was a mistake in Figure 1 as published. In the chemical structure of two intermediates in the biosynthesis of menaquinone a key hydroxy group was missing. The corrected Figure 1 appears below.

**Figure 1:**
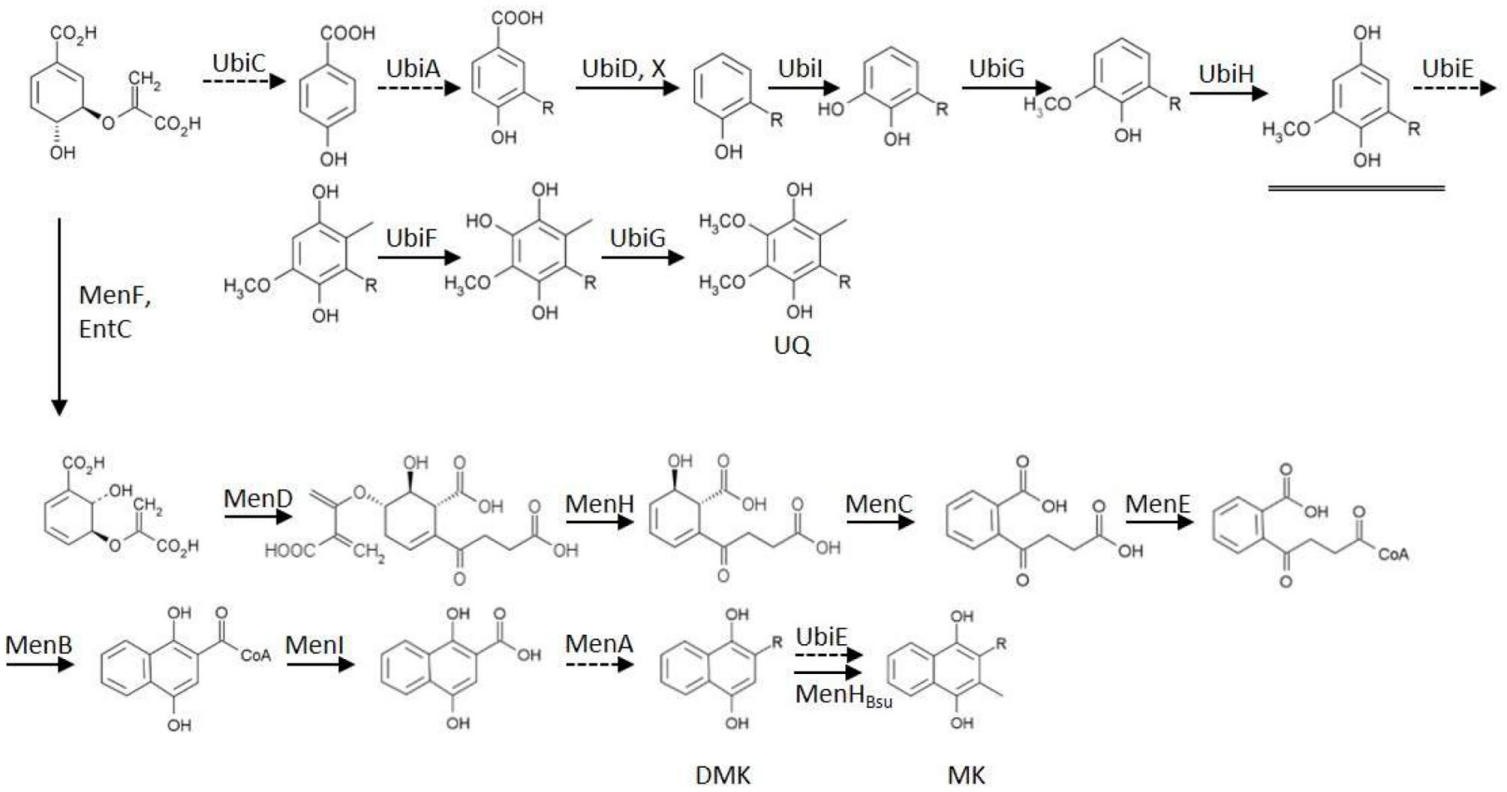
**Biosynthesis routes of the quinones of *Escherichia coli*, starting from chorismate**. Dashed arrows indicate enzymes deleted in mutants used in this study. The double arrow symbolizes the action of the introduced heterologous MenHBsu. The underlined intermediate C1-demethyl-C6-demethoxy-Q8 (DDMQ8) may accumulate in a ubiE mutant strain (for references: see text). Established bio-active quinones are indicated via their abbreviation. UQ, ubiquinone; DMK, demethylmenaquinone; MK, menaquinone; R, isoprenoid sidechain.

The authors apologize for these errors and state that this does not change the scientific conclusions of the article in any way. The original article has been updated.

