# Supplementary material for: All Three Endogenous Quinone Species of Escherichia coli Are Involved in Controlling the Activity of the Aerobic/Anaerobic Response Regulator ArcA
Source: Front Microbiol. 2016 Sep 7;7:1339. doi: 10.3389/fmicb.2016.01339 (PMC5013052; doi:10.3389/fmicb.2016.01339)

Supplementary Figure 1. **Plasmid maps of new constructs used in this study**. Plasmids were based on pDOC-K (Lee et al., 2009). A: pDOC-S-ubiCA. The *ubiCA* sites are flanked by I-SceI restriction sites, sizes are approximations. B: pDOC-K-yoeG-ParaMenH. The *yoeG* sites are flanked by I-SceI restriction sites, sizes are approximations.

A

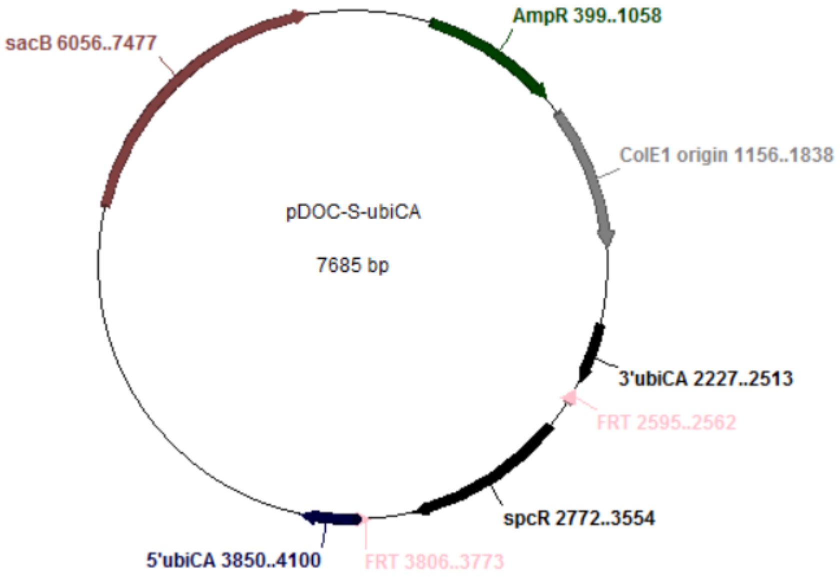

B

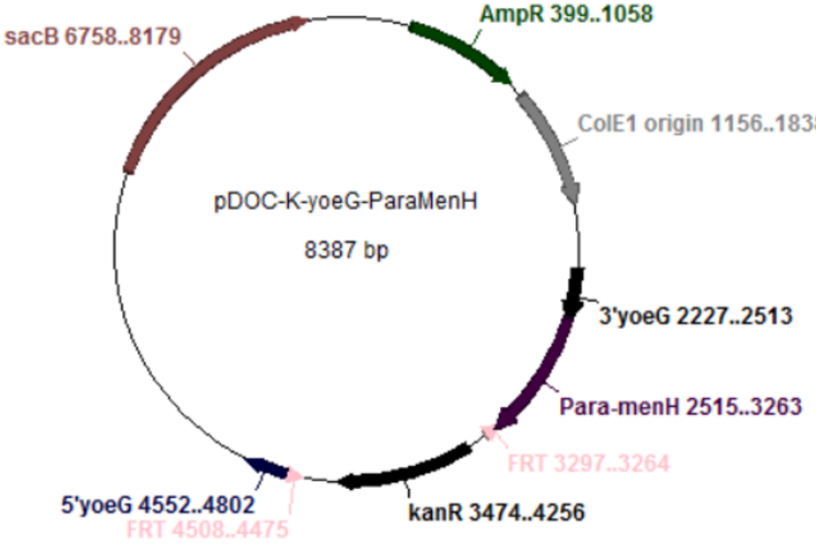

Supplementary Figure 2A-D. **Quinone pool redox state of *E. coli* MG1655.** Redox state (open figures) and concentration (closed figures) of all quinones in E. coli MG1655. A: All quinones together; B: UQ; C: DMK; D: MK. The bar above indicates N2 (gray) or air (white) sparging. Data based on the average of three biologically independent replicates. Error bars indicate standard deviation.

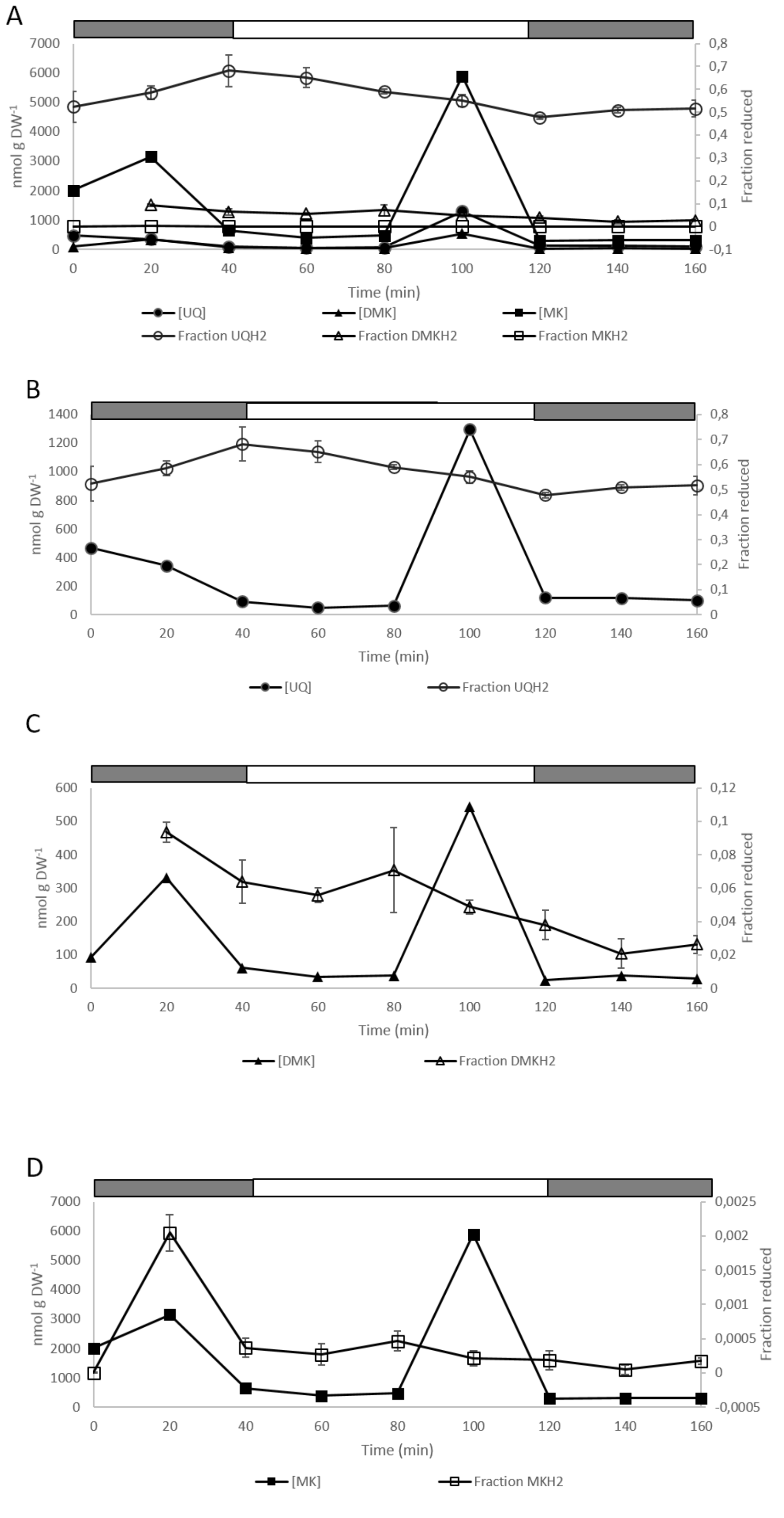

Supplementary Figure 2E-G. **Quinone pool redox state of *E. coli* UQ- (i.e. AV34), DMK- and MK-only mutant strains.** Redox state (open figures) and concentration (closed figures) of all quinones in E. coli MG1655. E: AV34 (UQ-only); F: DMK-only; G: MK-only. The bar above indicates N2 (gray) or air (white) sparging. Data based on the average of three biologically independent replicates. Error bars indicate standard deviation

E

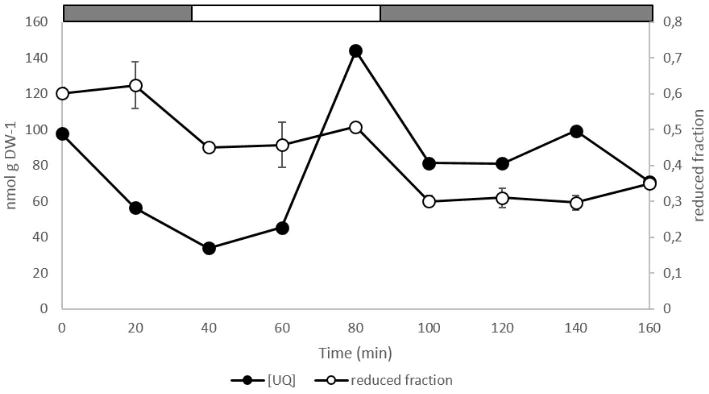

F

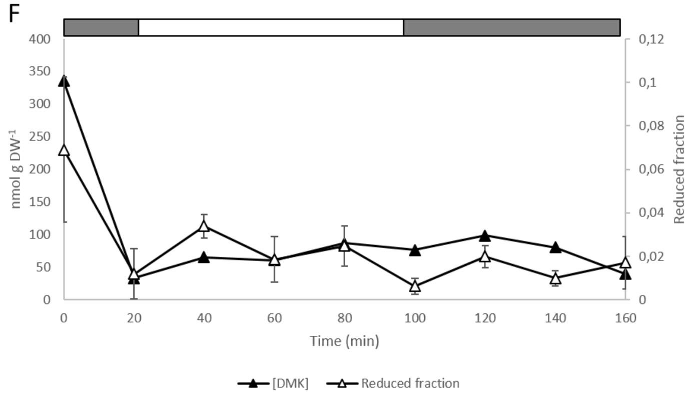

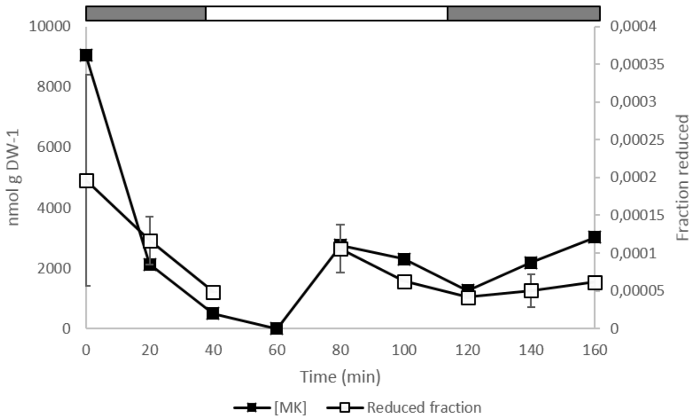

Supplementary Figure 3: **Exo-metabolite analysis of various *E. coli* strains**. A: Metabolites of *E. coli* MG1655; B: *E. coli* AV34; C: *E. coli* DMK-only; D: *E. coli* MK-only. The bar above each panel indicates sparging with N_2 (gray) or air (white). Data represent the average of three biologically independent replicates, error bars indicate the standard deviation.

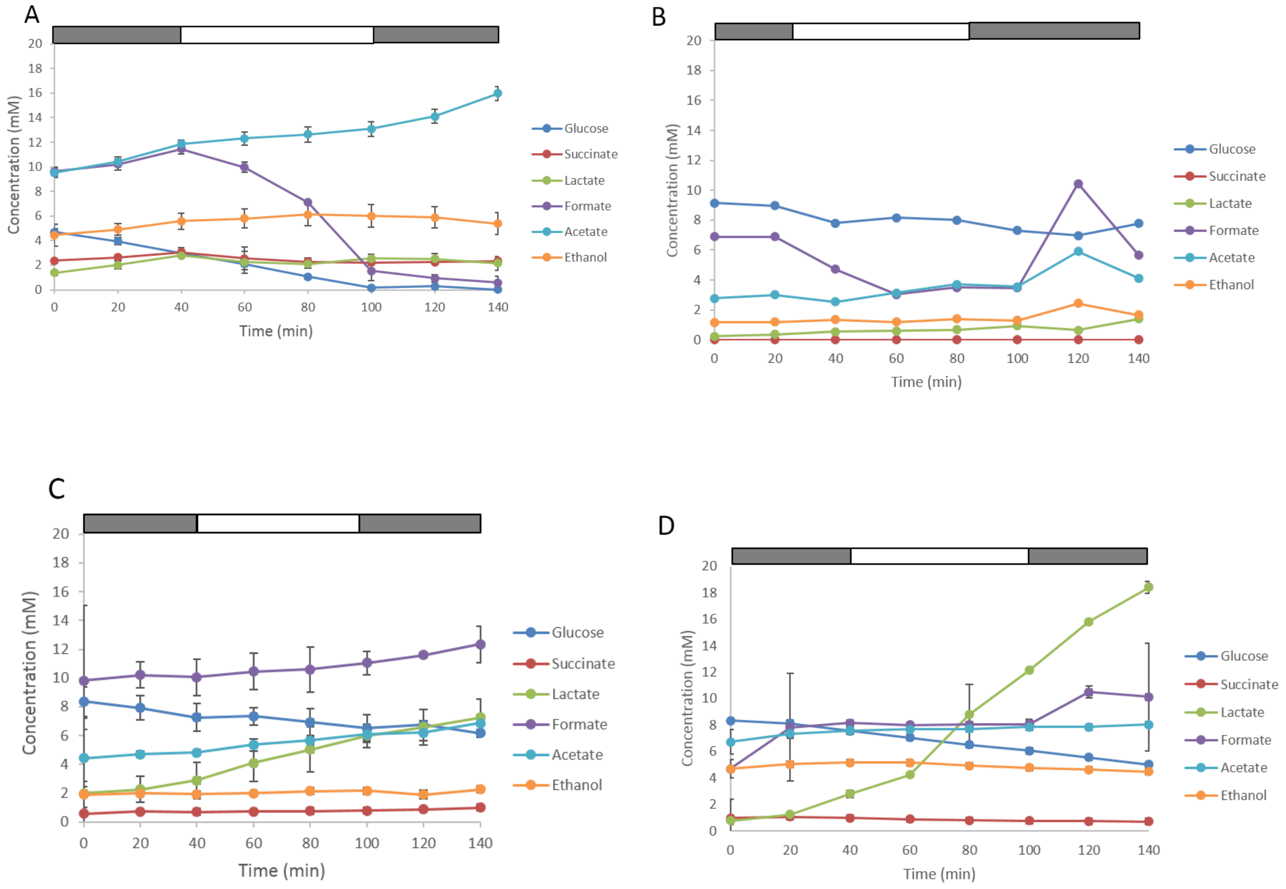

Supplement: Supplementary file 1 [file Data_Sheet_1.PDF]
